# Primary care practitioners’ and patients’ views on the benefits and challenges of remote consulting for anxiety and depression in UK general practice: a qualitative interview study

**DOI:** 10.1136/bmjopen-2024-093795

**Published:** 2025-06-08

**Authors:** Charlotte Archer, Louise Ting, David Kessler, Nicola Wiles, Katrina M Turner

**Affiliations:** 1 Population Health Sciences, Bristol Medical School, University of Bristol, Bristol, UK

**Keywords:** Anxiety disorders, Depression & mood disorders, QUALITATIVE RESEARCH, MENTAL HEALTH, Primary Health Care, Risk Assessment

## Abstract

**Abstract:**

**Objective:**

To explore primary care patients’ and practitioners’ views and experiences of remote consulting for common mental disorders (CMDs), to optimise their management in primary care.

**Design:**

Qualitative study using in-depth interviews and thematic analysis. A topic guide was used to ensure consistency across interviews. The interviews were audio-recorded, transcribed verbatim and analysed thematically. There was patient and public input throughout.

**Setting:**

Participants were recruited from general practices. Interviews were held by telephone or videocall between March 2023 and October 2023.

**Participants:**

We interviewed 20 practitioners and 21 patients.

**Results:**

Interviewees suggested benefits included convenience, increased anonymity and were easier for those feeling very low or anxious. Challenges included practitioners finding it hard to assess risk, which lengthened consultation duration or led to further contact, increasing practice workload and patients feeling anxious waiting for the practitioner to call. In-person appointments were viewed as important for initial consultations and providing a safe space. Continuity of care and practitioner training were identified as facilitators for telephone consultations, and both patients and practitioners identified training needs around how to deliver mental healthcare remotely.

**Conclusions:**

Practitioners should aim to offer continuity of care and in-person appointments when patients initially seek help. Remote consultations may not be more time or cost-efficient for individuals with CMDs as risk is harder to assess. There is a need to evaluate existing training on delivering remote consultations to identify whether remote mental healthcare is included or should be incorporated in the future.

**STRENGTHS AND LIMITATIONS OF THIS STUDY**

- Patient and public involvement was actively embedded in all stages of the research process, and participants had a wide range of sociodemographic and clinical characteristics.
- Patients were recruited if they already had anxiety or depression in their recent medical history. Therefore, this study does not capture the views of those who are yet to seek help for their mental health.
- Participants were self-selecting in terms of being individuals who responded to the research invitation. As such, those interviewed may have been individuals who had strong preferences for or against remote consultations.

## Background

 Anxiety and depression are managed in primary care with general practices (GPs) best placed to offer the continuity of care that these conditions often require.^1^ During the COVID-19 pandemic, most primary care consultations switched to remote delivery, usually telephone or message-based platforms (termed e-consults), or occasionally by videocall.^2^ Since then, the proportion of in-person consultations has not increased back to prepandemic levels, with around one-third of appointments delivered remotely.^3 4^

While remote consultations may provide improved access to healthcare,^5^ some patients may not have a private space to discuss sensitive issues, and some may consider remote technology inappropriate for sharing health concerns.^6 7^ A mixed-methods study—on how clinicians dealt with the implementation of remote consulting during the COVID-19 pandemic—found that GPs and nurses considered high levels of remote care challenging, but that they could be useful for the ongoing management of chronic conditions.^2^

Previous studies in primary care have not explored the impact of remote consultations on those with common mental disorders (CMDs), that is, anxiety and depression. Research in secondary mental healthcare suggests patients and practitioners regarded remote consultations as necessary during the pandemic, but found the loss of ‘safe space’ and non-verbal cues difficult.^7 8^ An evidence briefing on the impact of remote consultations on personalised care stated that the visual signals, often noted during mental health assessment, were lost over the telephone or with e-consults.^9^ Conversely, CMDs can impact an individual’s ability to attend or discuss problems in-person,^10^ and patients with CMDs may find remote care more accessible and acceptable compared with attending in-person.

Much of the existing evidence on remote consulting was either conducted prior to the pandemic or is focused across all conditions and not specific to mental health. Given that around 40% of primary care consultations involve mental health,^11^ and remote consulting is now embedded within general practice, it is important to reflect on when it is best used to ensure safe and effective management of CMDs. This study explored primary care patients’ and practitioners’ views and experiences on the benefits, challenges and facilitators of remote consulting for CMDs to inform its use in general practice.

## Method

Ethical approval was provided by South West - Frenchay Research Ethics Committee (REC reference: 22/SW/0136).

### Recruitment and sampling

Practitioners and patients were recruited through GP practices in Bristol and the surrounding areas. The West of England Clinical Research Network (CRN) informed these practices about the study and passed details of interested practices to the research team. The research team selected practices to support study recruitment based on their varying deprivation deciles and sociodemographic characteristics of their patient populations.

Practitioners were informed about the study by practice managers. Eligible practitioners were those working in to take part in the study and providing mental healthcare to patients with anxiety or depression. Practitioners were excluded if they did not meet both of the inclusion criteria. Practitioners willing to be interviewed emailed the research team their contact details, clinical role and demographic details (eg, age and gender). This information, alongside knowledge of their practice, was used to purposively sample practitioners of varying roles, age and gender. Initially, the research team aimed to recruit GPs and practice nurses, as they both have a role in supporting primary care patients with mental health problems. However, as data collection progressed, it became apparent that many practices had other allied health professionals (AHPs) who also provided mental healthcare. Therefore, the study’s inclusion criteria were expanded to include AHPs, including practice pharmacists and well-being coaches.^12^

Patients were invited for interview via their GP practices. Practices identified potential participants through searches of their practice electronic databases. Eligible patients were aged ≥18 years, with a current diagnosis of anxiety and/or depression, who had consulted their practice for their CMD either remotely or in-person, in the past 6 months. Patients were excluded if they had bipolar, schizophrenia, personality disorder, dementia or substance (alcohol/drug) misuse in the past year. Practices posted an invitation letter and information sheet to potential participants. Those interested in participating returned response forms (*contact details, sociodemographic (age, gender, ethnicity), CMD (diagnosis, duration, past episodes and medication) and mode of mental healthcare access (in-person, telephone, video and/or e-consult*)) using reply paid envelopes. This information, alongside their practice’s deprivation decile, was used to purposively sample individuals varying in these attributes.

### Data collection

Interviews were conducted by CA (an experienced qualitative researcher) by telephone or videocall. Verbal consent to participate was audio-recorded immediately prior to interview. Two topic guides (one for practitioners; one for patients) were used to ensure consistency across the interviews (online supplemental file 1, 2). The guides were developed in parallel and were based on the aims of the study, relevant literature and discussions with the research team and patient and public involvement (PPI) contributors.

Practitioners and patients were asked about their views and experiences of remote consultations for CMDs, the benefits, challenges and facilitators of these consultations and when they thought in-person appointments were preferable to remote modes of consultation. After each interview, patients and practitioners were asked to complete a brief questionnaire (employment status, education, marital status), the information from which was used to describe those interviewed and considered during data analysis. All interviewees were financially reimbursed to thank them for their time.

### Data analysis

Interviews were audio-recorded using an encrypted voice recorder, professionally transcribed, fully anonymised and checked for accuracy. Data collection and analysis proceeded in parallel so that analytical insights from early data collection could shape later data collection and so that data collection would end when data saturation had been reached, that is, no new themes were identified in later interviews. Thematic analysis was undertaken following the steps defined by Braun and Clarke,^13^ to allow comparisons to be made within and across patient and practitioner interviews.

Each data set was analysed separately. Two investigators (CA and KT) read and re-read a sample of practitioner and patient transcripts to identify possible codes and then met to compare and discuss their coding and interpretation of the data. Two preliminary coding frameworks (one for practitioners; one for patients) were developed in parallel, to ensure codes common to both were included in each. Each coding frame was revised as new codes were identified in later transcripts, and previously coded transcripts were re-coded where necessary. All transcripts were electronically coded in NVivo (V.12) so that data relating to each code could be easily extracted. To aid interpretation of the data, extracted data were summarised in a table where rows represented interviewees and columns specific codes. The tables were then read and re-read to identify key themes and deviant cases. Once each data set was fully analysed, the views and experiences of practitioners and patients were compared, allowing similarities and differences to be identified.

### Patient and public involvement

A PPI co-investigator (LT) provided input at every stage of the research. LT and two PPI contributors, all of whom had lived experience of anxiety or depression, met with CA and commented on initial ideas for the study and advised on patient-facing materials and topic guides. Seventeen months later, CA, LT and six PPI contributors discussed the study findings via an online meeting and written feedback. All felt that the results were important and relevant and highlighted key messages and avenues for dissemination.

## Results

Twenty practitioners from five practices were interviewed between March and October 2023 (table 1). Interviews lasted, on average, 28 min (range: 14–38 min). Over half of the practitioners were female (n=12, 60%), and the mean age was 43.2 years (SD=9.0). Those interviewed had been working in general practice between 1 and 36 years, and all were regularly using telephone and e-consults. Most (n=18) had experienced video consulting (for any indication), but only three AHPs were regularly using video for mental health consultations.

**Table 1:** Characteristics of practitioners interviewed and practice deprivation deciles

*Characteristic*		*N*	*%*
*Gender*	*Female*	12	60
	*Male*	8	40
*Age, years*	*20–29*	1	5
	*30–39*	8	40
	*40–49*	7	35
	*50–59*	3	15
	*60+*	2	10
*Ethnicity*	*White*	16	80
	*Mixed*	1	5
	*Asian*	3	15
*Role in practice*	*GP partner*	6	25
	*Salaried GP (including GP trainee*)	9	45
	*Allied health professional*	5*	25
*Mental health qualifications*	*Yes*	5	25
	*No*	15	75
Practice deprivation score†	*1–5*	7	35
	*6–10*	13	65

* n=3 pharmacists, n=1 nurse and n=1 well-being coach

† Deprivation score for the practice patient population where one indicates the most deprived patient population and 10 the least deprived. Taken from the National General Practice Profiles website^33^ which calculated scores based on the 2015 English Indices of Deprivation.^34^

GP, general practice.

Twenty-one patients from four practices were interviewed between April–October 2023 (table 2). Interviews lasted, on average, 33 min (range: 13–45 min). Over half were female (n=13, 65%), and the mean age was 49.7 years (SD=16.9). All had a diagnosis of anxiety, depression, or both anxiety and depression. Most had experience of consulting in-person (n=15) and by telephone (n=18) about their mental health, and over half (n=12) had used e-consults. None had experienced consultations by videocall for their mental health.

**Table 2:** Characteristics of patients interviewed and practice deprivation deciles

*Characteristic*		*N*	*%*
*Gender*	*Female*	13	61.9
	*Male*	8	38.1
*Age, years*	*18–29*	3	14.3
	*30–39*	4	19
	*40–49*	3	14.3
	*50–59*	3	14.3
	*60–69*	5	23.8
	*70+*	3	14.3
*Ethnicity*	*White*	15	71.4
	*Mixed*	3	14.3
	*Black*	1	4.8
	*Asian*	2	9.5
*Highest educational qualification*	*Higher diploma or degree or equivalent*	7	33.3
	*General Certificate of Secondary Education (GCSE), O-level, A-level or equivalent*	11	52.4
	*No formal qualifications*	3	14.3
*Marital status*	Married/living as married	11	52.4
	Single	7	33.3
	Divorced	3	14.3
*Employment status*	*In paid employment*	12	57.1
	*Retired*	5	23.8
	*Unemployed due to ill health*	4	19.0
*Diagnosis*	*Anxiety*	6	28.6
	*Depression*	3	14.3
	*Mixed anxiety and depression*	12	57.1
*Duration of current episode*	*2 weeks to 6 months*	4	19.0
	*6 months to 2 years*	5	23.8
	*2 to 5 years*	5	23.8
	*5 to 10 years*	4	19.0
	*>10 years*	3	14.3
*Past anxiety or depression*	*Yes*	15	71.4
	*No*	6	28.6
*Currently taking antidepressant medication*	*Yes*	16	76.2
	*No*	5	23.8
Practice deprivation score*	*1–5*	8	38.1
	*6–10*	13	61.9

* Deprivation score for the practice patient population where one indicates the most deprived patient population and 10 the least deprived. Taken from the National General Practice Profiles website^33^ which calculated scores based on the 2015 English Indices of Deprivation.^34^

**Figure 1:**
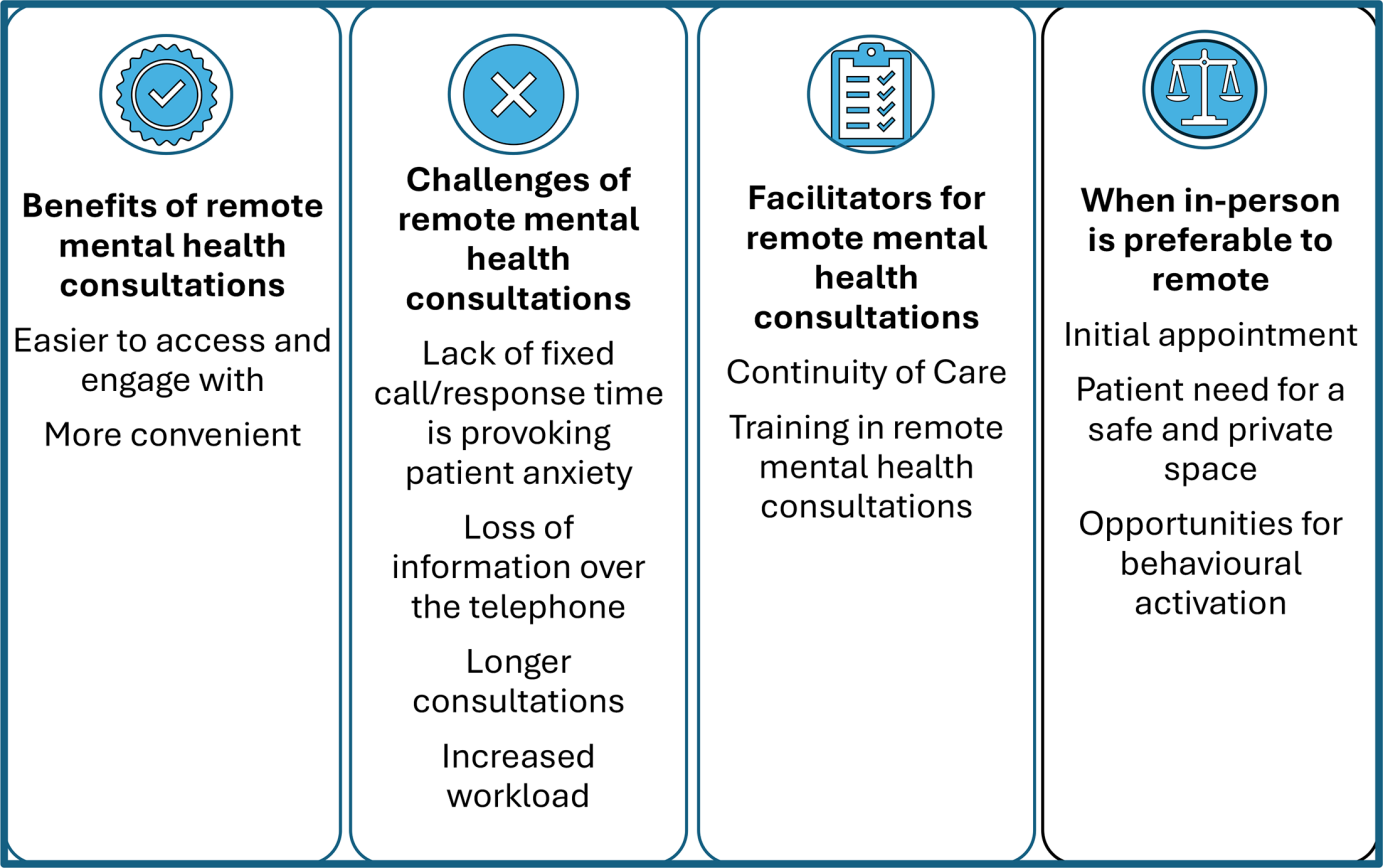
Key areas for the benefits, challenges and facilitators of remote consulting for anxiety and depression.

Within each data set, 11 themes were identified that could be placed under four headings (figure 1). As most of these themes were common to both groups of interviewees, below, findings from practitioners and patients are presented together. Most of the themes related to telephone consulting, reflecting that this was the mode of remote consulting interviewees had most experience of. Themes related to e-consults are those that are specific to CMDs, rather than generic for all health conditions.

### Benefits of remote mental health consultations

#### Easier to access and engage with

Practitioners and patients commented that there were some groups for whom remote consulting was more suitable, because they felt unable to leave their home due to feeling anxious or depressed or felt more comfortable discussing their symptoms from home.

‘*When people are anxious and depressed…going to the GP surgery for a lot of people…can make people quite anxious…when they are at home…they can tell you more*. GP 11

Both groups explained that telephone appointments could be easier for some patients because they did not have to ‘make eye contact*’* (Patient 15) or ‘put on a show…get dressed…be seen as though they are alright*’* (GP 13).

The anonymous nature of a telephone consultation was also mentioned as facilitating disclosure of sensitive information.

*I know there is somebody there, but I do not have to see them…[their] expression…sometimes I feel like when I talk about it [in-person], even though I know she is not, I feel like I am being judged…on the phone I do not feel that way…I cannot see nobody so I feel confident to talk*. Patient 18

#### More convenient

Practitioners and patients thought that, overall, e-consults and telephone worked well for follow-up appointments when conversations could be short, transactional and on specific issues such as medication. They also noted they could be more convenient for patients, with quicker access to healthcare by telephone.

*[Telephone] is more convenient for them…they can just pop out [to take the call] instead of coming up to the surgery*. AHP 4

*I could have asked for a face-to-face one, but it was more convenient [to have a telephone appointment]…and because it was just a follow up and everything else seemed to be going alright, phone appointment was fine*.” Patient 13

### Challenges of remote mental health consultations

#### Lack of fixed time for telephone call/unknown response time provokes patient anxiety

Although patients said follow-up telephone appointments were acceptable, they explained they had to plan their day around the call in case they missed it as there was no fixed time. Many patients described waiting for this call as anxiety-provoking.

*With…mental health problems…[you] become incredibly hyper-anxious…you are told somebody is going to ring…between nine and five…that is no good to me…it elevates anxiety levels…waiting and waiting to speak to somebody*.” Patient 12

This also applied to e-consults, whereby patients described uncertainty around when or what type of response they would get.

*Not knowing how long it is going to take…you feel, right, I have been waiting all day for them to get back to me…it would just be nice…a way of knowing…when approximately when they would get back to you…you are a bit anxious anyway*.” Patient 7

#### Loss of information over the telephone

Practitioners and patients suggested there could be a difference between what people said on the telephone and their body language, with patients aware that they might be more likely to withhold information compared with consulting in-person. Due to the lack of non-verbal cues, practitioners said that they found it harder to assess risk over the telephone and had to listen carefully to *how* people said things.

*If you say, ‘do you feel suicidal’…the ones who actually do commit suicide will say ‘no’ on the phone. But if they are face-to-face, they might say ‘no’ but it is just the way they are looking away from you that it might make you probe into it a bit more*. GP 24

*Seeing someone in-person is a lot different to…just speaking on the phone…it could just be easier to hide what is going on or they might not feel that they care that much, like ‘oh we will just speak on the phone*’. Patient 21

A few GPs reflected that, because they were more concerned about underestimating risk on the telephone, it might mean that they were more likely to prescribe medication.

*Certain sorts of patients, you…probably start prescribing more medication [on the telephone] than you normally would…you do not feel as comfortable with your risk assessment…I end up feeling like I need to give them some treatment just in case*. GP 19

In contrast, some of the AHPs said they wanted to ‘see’ their patients before they would initiate psychotropic medication.

*If they’re wanting to start antidepressants, I will not do that unless I am able to have a videocall or face-to-face appointment…it is for my prescribing safety and also the patient’s safety*. AHP 2

Patients and GPs suggested that when appointments about the CMD repeatedly took place over the telephone, it could feel like the calls were not getting to the root of the problem.

*For me personally, face-to- face is a lot more easier than telephone calls… I feel like it could resolve the situation a bit quicker* Patient 14

*You start to feel like you have become their intervention…rather than you are actually treating them…you become their person they chat to on the phone to make them feel better for that moment…you don’t necessarily get down to the grit of it*.” GP 19

There was a sense that telephone calls were less comprehensive without the visual cues, so skilled questioning to elicit information on aetiology and severity of the CMD, alongside conveying understanding and reassurance, was pertinent when using this mode.

#### Longer consultations

Practitioners explained that although there was a perception that telephone appointments took less time to deliver, actually, they could ‘pick up on more things easily’ (GP 15) in-person and felt they needed to cover ‘safety-netting’ more thoroughly on the telephone.

*Telephone probably takes longer…you can make assumptions when somebody is in the room…self-care…eating…that is a quick judgement whereas you may have to ask quite detailed questions on the phone around that. And the sort of safety netting side…is somebody safe, when are we going to follow up…that bit takes longer*.” GP 23

In contrast, patients described how they thought telephone was quicker for GPs than in-person appointments. This was a reason for patients suggesting the consultation was remote, as they were aware GPs were busy and did not want to ‘overburden’ (Patient 17) them.

*I said ‘look it is fine, I really do not need to come to see you, we can just talk on the phone’…I just felt I did not want to waste any more of her time*. Patient 1

#### Increased workload

Patients and practitioners said that when an e-consult was submitted, the patient would usually get a telephone call back. Most practitioners explained they would rarely do a CMD consultation just by e-consult, as risk was difficult to assess due to insufficient or unclear written information provided by the patient. In addition, some patients explained they often did not seek help until they reached a crisis, indicating a more urgent need for an appointment. Practitioners said that they did not want to keep patients who felt low, waiting. Hence, such e-consults would be followed up promptly with a telephone call, with practitioners noting this increased their workload.

*We often end up phoning them anyway…we have to over-prioritise what is actually fairly low-level mental health because you do not want to leave someone who is saying they are so low…in case they go and harm themselves. AHP 3*

### Facilitators for remote mental health consultations

#### Continuity of care

Practitioners and patients reported that continuity of care was particularly important for telephone calls about CMDs. Practitioners noted its value in assessing risk remotely, knowing the normal ‘tone…or speed of their voice’ (GP 14). Similarly, patients said they found it easier to talk to a practitioner who already knew their background; there was a sense that patients found talking about mental health difficult.

*Even though I am a chatty person…when it comes to things like that, I am very wary who I talk to*.” Patient 18

However, many patients said it was very difficult to book telephone appointments with the same GP, but continuity of care was easier with an AHP.

#### Training in remote mental health consultations

Practitioners said they had not received any training on conducting mental health consultations remotely, but many felt this was needed.

*Over the phone…it is quite a different skill…those extra considerations…who is in the room…can they actually talk right now…it is not necessarily a safe space for a consultation…I think [we] definitely need more training in it*. GP 17

GPs who had been working for longer were more comfortable without the training, but thought there was a need for in-person experience for newly qualified practitioners before they started telephone CMD consultations.

*People do need to see a lot of face-to-face at the beginning of their career…they can pick up on what can and cannot be safely managed over the phone, they [need to] learn that.* GP 11

Practitioners suggested a need for training on risk management and how to convey empathy when consulting remotely, as they found risk harder to assess, and the lack of non-verbal cues meant they had to demonstrate empathy using their voice.

*All the cues are verbal, you have got to put empathy within your voice…you may have to just say ‘oh that’s terrible’…but you do have to shut up as well…you must let people speak and give them a good opportunity to speak before you ask any particular closed questions…the skills, it is different*. GP 21

Similarly, patients also talked about the need for practitioner training in conveying empathy over the telephone. They viewed empathy as a key facilitator in encouraging disclosure around CMDs, leading to a more accurate risk assessment, as well as the need for practitioners to develop active listening skills, so they did not interrupt the patient but listened carefully and reflected back to them.

*You can tell the ones who have had some [remote] mental health training…more patient…empathetic…understanding…the ones who I do not think necessarily have had the training seem rushed and when you get upset…[they] do not know what to do…you can tell it in their voice….they are just very quiet and it is as if there is nobody there [on the telephone]*. Patient 7

Some patients also thought practitioners needed training in knowing when to ask patients to attend in person, even if the patient’s preference was for telephone.

‘*[They need to know] whether it is ok, is this telephone appointment or is this somebody who needs a face-to-face…they definitely need the mental health training to pick up on that*. Patient 7

### When in-person is preferable to remote

#### Initial appointment

Many practitioners and patients said they preferred an in-person consultation for initial CMD appointments, due to the loss of information over the telephone, as outlined earlier. Patients also said they preferred in-person if they were feeling very low or anxious, as such appointments were more personal because they could see their practitioner’s reactions and they felt it was easier to explain how they were feeling because they thought they were more likely to be believed and that the practitioner was more likely to listen to them.

*Going in makes such a difference….you can see when they are looking at you, they really believe you…they knew I was not pretending.” Patient 1*

#### Patient need for a safe and private space

Practitioners and patients said that one or two in-person appointments were important for establishing rapport and providing a safe space to share sensitive information. Patients noted a safe space also was important when talking about how their mental health might be affecting their relationships with others, and disclosing information that might make them feel vulnerable.

*If I wanted to talk about how it was affecting my relationships and things, I would probably feel more comfortable knowing I was in a safe and confidential place…confiding in someone is such a big thing, on the phone would leave me feeling very vulnerable afterwards*. Patient 15

GPs and patients thought that some older patients were reluctant to bring up symptoms of CMDs, but might be more likely to share them after discussing physical health during an in-person appointment, where they had more opportunity to open up.

*I would say ‘I need to see the doctor’, they might then ask what is happening…I will say ‘oh, you know, it is like something else’ and then when I see her then she realises that it is not something else, I am not feeling mentally well….I usually go in there with a physical ailment of some sort*. Patient 17

#### Opportunities for behavioural activation

Some practitioners suggested that for patients who were anxious to leave home, attending in-person could be part of encouraging the patient to take control of their mental health and set achievable goals. This point was primarily made by the AHPs interviewed and by those who had patients with a chronic CMD with whom they had developed a good doctor-patient relationship during long-term remote care.

*I just frame that as a possible goal…might be to come and see me and how we might work getting them to come in and see me. AHP 1*

## Discussion

### Summary

Practitioners and patients view remote consultations as improving access to care for patients with CMDs, but appropriateness depends on patients’ preferences and circumstances, previous contact and symptom severity. Remote consultations can be easier for patients who are feeling very low or anxious and can be more convenient, particularly for follow-up appointments. However, practitioners say that risk is harder to assess remotely due to the loss of information, and some are more likely to prescribe medication if they were not confident in their ability to assess patient risk when consulting remotely. Patients state that they may withhold information, find it harder to disclose sensitive information or have concerns about not being believed or their distress recognised. For these reasons, they note that in-person appointments are important for initial consultations and to provide a safe space to facilitate disclosure.

Remote consultations may provide patients with quicker access to care, and hence patients may accept this offer as they are aware of the pressures on general practice. However, patients find waiting for a telephone call from the GP difficult because of the wide calling window and are often uncertain how quickly they will receive a response to an e-consult. While remote consultations may improve access to care, practitioners report that remote consultations for mental health problems are not necessarily more time-efficient, with patient contacts via e-consult often needing a follow-up telephone call.

Patients and practitioners view continuity of care and training in remote consulting for mental health problems as facilitators. However, they identified training needs around identifying risk in the absence of non-verbal cues, developing skills in active listening, conveying empathy and encouraging patient disclosure.

### Strengths and limitations

PPI was embedded in all stages of the study, thereby influencing every stage of the research. We recruited interviewees with a wide range of sociodemographic and clinical characteristics. However, we recruited patients who had anxiety or depression in their recent medical history. This study, therefore, does not capture the views of those who have not yet sought help. Likewise, patients and practitioners were self-selecting in terms of being individuals who responded to the invitation, which stated the research was focused on remote methods (telephone, video, e-consult) for consulting in general practice, and that the interviews were being held by telephone or videocall. It is possible that those interviewed were individuals who had strong preferences for or against remote consultations and were comfortable being interviewed remotely. Patients and practitioners had little experience of receiving or providing care by videocall, thus limiting our understanding of the benefits, challenges and facilitators of this mode of consultation. Future studies could seek to interview primary care patients who have used videocalls for CMDs. That said, most practices do not offer video consultations frequently, so it would be challenging to seek in-depth views on this mode.^14^

### Comparison with existing literature

Concerns about the use of telephone and video consulting in primary care highlight missed diagnoses, challenges to therapeutic relationships,^15^ difficulties in doctor-patient interactions, feeling rushed^16^ and less engagement with services.^17^ Our study supports some of these, suggesting risk is more difficult to assess over telephone, potentially taking longer and challenges in establishing patient-practitioner rapport. Practitioners in our study also noted that e-consults may not be that efficient, often leading to additional contacts, and that telephone appointments may take longer than in-person consultations. This is in line with a systematic review investigating the impact of telephone and video primary care consultations—for any indication—finding that they may increase the need for additional GP appointments compared with in-person,^18^ and with wider literature around mental health appointments taking longer.^8 19 20^ It is clear from our study and others^14^ that videocalls are not often used in general practice.

Many of our interviewees said that remote modes increased access to care, and this supports findings by NHS England.^5^ Unfortunately, increased provision of care by practitioners does not always equate to increased engagement by those accessing the care, with some patients sharing their experience of limiting how much information they disclosed over the telephone, potentially due to the transactional nature and not wanting to take up practitioners’ time. A recent briefing highlights that it is important to consider access to healthcare as more than just provision of appointments; it is also about broader factors, such as health literacy, barriers to reach services and how people identify themselves as candidates for medical attention.^21^

There is also a contradiction in our data between the importance of continuity of care yet the need to be able to respond rapidly in situations where risk is high. This is challenging to resolve as patients’ ability to see the same practitioner is likely dependent on service delivery pressures and the urgency of the problem, which is often closely linked to risk, further compounding this contradiction.^22^ Some practitioners in our study suggested they might be more likely to prescribe over the telephone if they were concerned about risk. This is not in line with analyses conducted prior to the pandemic using secondary care electronic health records, which did not find an increase in psychiatric prescribing.^23^ However, there is some evidence that remote consultations have higher doses of anxiolytics^24^ and increased antibiotic prescribing rates,^25^ suggesting mode may be a factor in prescribing decisions.

Most interviewees preferred in-person appointments for initial consultations for a CMD, and a survey of patient experiences during the pandemic—for all indications—also makes this recommendation.^26^ In our study, in-person consultations at the practice were considered key in providing a safe and private space to discuss concerns. This is consistent with other research exploring the role of place in GP consultations.^27^ Not all patients are able to access appropriate private spaces for remote consults, and this can negatively impact their experiences of care, thus creating inequities. It was noted that such spaces are particularly important for those discussing sexual or mental health problems.^27^ Greenhalgh *et al*^28^ note that staff training for remote consultations has not kept pace with NHS plans. Although there are training resources covering remote risk assessment and rapport,^29^,^31^ there is a lack of training specific to remote primary mental healthcare. Interviewees in our study suggested that mental health consultations may be more complex than appointments for physical health, requiring greater skill to encourage disclosure and assess risk. Other interviews with patients and practitioners have identified training as a facilitator in implementing remote mental health consultations during the pandemic,^32^ although practitioners in our study said they had not received such training.

### Conclusions

This study aims to understand primary care patients’ and practitioners’ views and experiences on the benefits, challenges and facilitators of remote consulting for CMDs. Findings suggest that it is important to offer in-person appointments, especially when patients initially seek help for anxiety or depression, and are unknown to the practitioner. Ideally, practices should provide a narrower window for a telephone call, as many patients find waiting for the telephone call difficult. The challenges in assessing risk remotely and patients’ hesitancy to discuss their mental well-being mean that it is important for practitioners to convey empathy, ask questions that encourage disclosure and demonstrate active listening. Some practitioners may benefit from training in this area. There is a need for future research to review existing training, determine whether it needs to be expanded to cover mental health consultations more specifically and, if so, consider how best to deliver it with busy practitioners with subsequent evaluation.

## Supplementary material

10.1136/bmjopen-2024-093795 online supplemental file 1

## Data Availability

Data are available upon reasonable request.
