# Supplementary material for: Primary care practitioners’ and patients’ views on the benefits and challenges of remote consulting for anxiety and depression in UK general practice: a qualitative interview study
Source: BMJ Open. 2025 Jun 8;15(6):e093795. doi: 10.1136/bmjopen-2024-093795 (PMC12161304; doi:10.1136/bmjopen-2024-093795)
Supplement: online supplemental file 1 [file bmjopen-15-6-s001.docx]

**Primary care practitioners’ and patients’ views on the benefits and challenges of remote consulting for anxiety and depression in general practice: A qualitative interview study**

**Supplementary files**

***Supplement 1: Practitioner Topic Guide***

**Consulting remotely**

1. To start with, please can you tell me about which modes of remote consultation you have used for patients with anxiety or depression in the last year? For example, e-consults, telephone calls or videocalls.
   1. Does your practice have any specific guidance in relation to when or how these methods should be used, either in general or for mental health consults? If so, what?
   2. [*If no*] What is your understanding of the practice requirements or limitations in terms of phone or in-person consults?
   3. What informs which mode you use for anxiety and depression?
   4. Which have you used the most and why?
   5. Prior to the pandemic, had you held consultations remotely before? If so, how and why?
      1. What training did you have when you started using remote consults for mental health, if any? What did you learn? Did it feel sufficient?
2. Does any particular mode take longer than the other forms when discussing mental health?
   1. How do you manage building a rapport with patients over the telephone or video? Do you do anything different compared to in-person?
      1. What impact does this have, if any?
   2. Is there anything you would do during an in-person consult, that you can’t or don’t do during a telephone or video consult? If so, what?
      1. What impact does this have, if any?
   3. Do you have a preference for in-person or telephone/video consults? Why?

**Types of remote consultations**

1. What do you think are the benefits and challenges of using e-consults for patients with anxiety and depression?
   1. How well do e-consults work for those consulting for anxiety or depression for the first time? How well do they work for follow up appointments?
   2. What do you do differently for a mental health e-consult, compared to physical health, if anything?
   3. What do you think are the benefits are of using e-consults are, for the practice?
2. What do you think are the benefits and challenges of using e-consults for patients with anxiety and depression?
   1. How well do telephone appointments work for those consulting for anxiety or depression for the first time? How well do they work for follow up appointments?
   2. In what other circumstances do you think they do/don’t work so well, compared to in-person appointments?
   3. What you do differently for a mental health telephone consult, compared to physical health, if anything?
   4. Do patients get a set appointment call time, or a calling window? If a calling window, why not a set time?
3. What do you think are the benefits and challenges of using e-consults for patients with anxiety and depression?
   1. How well do videocalls work for those consulting for anxiety or depression for the first time? How well do they work for follow up appointments?
   2. In what other circumstances do you think they do/don’t work so well, compared to in-person appointments?
   3. What you do differently for a mental health video consult, compared to physical health, if anything?

**Future use/training and support**

1. So thinking about how remote consultations should be used going forward, how do you think they should be used for patients with anxiety and depression in the future?
   1. What type of patient do you think would be most suited to using remote consultations in this way? What about the clinical factors?
2. Do you think specific training or support is needed to deliver mental health consultations in this way? If so, what do you think is needed?
3. Finally, do you have any other comments, related to remote consultations for anxiety and depression, or remote consultations more generally?

***Supplement 2: Patient Topic Guide***

**The individual**

1. So please can you tell me a bit about yourself, for example, your age, who you live with, and what you do?
2. Can you now tell me a bit about your mental health? What has it been like for you, what symptoms do you experience and how long have you had them?

**Their practice**

1. How far away is your practice, and is there is a GP you normally see?
2. What they think about your practice? How long have you been registered with them?
3. When needing an appointment what do you do? e.g. call the practice and speak to a receptionist
   - 1. Are you able to ask for an in-person appointment or are you offered a choice?
     2. If you aren’t offered a choice, do you feel like you would be comfortable asking for an in-person appointment? If yes, why, if not, why not?

**Use of different types of appointments**

1. In the past six months, have you discussed your mental health with a GP or nurse? Please can you tell me who you first spoke with, what the appointment was for/how did the subject of mental health come about, and what type of appointment you had? For example, either in-person, telephone, videocall or an e-consult.
   1. Was that the first time you had discussed your mental health with that practitioner?
   2. In terms of how the appointment was held, i.e. in person or by telephone etc, can you remember why you had that type of appointment? Was it your preference or what you were offered by your practice?
   3. How well did that type of appointment work for what you wanted to discuss?
   4. What happened doing that appointment? What was discussed and what decisions were made?
   5. If that appointment had been [in-person/telephone] instead, how would that have felt?
2. What happened after that appointment? Did you have further contact with that practitioner, either by telephone, videocall e-consult or in-person? ***[If yes return to question 2B, if contact with a different practitioner, move to 3A, if none at all move onto question 5].***
   1. *[If yes to other practitioner]* Please can you tell me who was it with, what was it for, and what type of appointment you had?
   2. Was that the first time you had discussed your mental health with that practitioner?
   3. Again, why did you use that type of appointment? Was it your preference or what you were offered by your practice?
   4. And again, how well did that type of appointment work for what you wanted to discuss?
   5. What happened doing that appointment? What was discussed and what decisions were made?
   6. If that appointment had been [in-person/telephone] instead, how would that have felt?
3. What happened after that appointment? Was there any further contact with your GP or nurse, either by telephone, videocall e-consult or in-person? ***[If yes repeat question 3/4, if no move onto question 5].***
4. Comparing the different appointment types you have experienced [i.e in-person and telephone etc], which did you feel most comfortable with? Which felt most appropriate for the reason you wanted to speak with a GP/nurse?
   1. Did using [video/telephone/e-consult] change your views on using it as an alternative to in-person appointments? If so, how?

**Advantages/disadvantages of different types of appointments**

1. Do you know if your GP practice offers e-consults? Is there a particular time or situation in which you would or wouldn’t use e-consults for your mental health? If yes, why?
   1. Is there anything that would done, or introduced, what would make e-consults more acceptable or accessible for you to use?
2. Is there a particular time or situation in which you would or wouldn’t use telephone appointments for your mental health? If yes, why?
   1. How important is it to have the same practitioner/GP each phone call? Does it make easier to discuss your mental health?
      1. [*If important*] How long would you wait to speak to your own GP?
   2. If you really wanted to be seen in-person, how long would you be willing to wait for that before you had a telephone call instead?
   3. Is there anything that would done, or introduced, what would make telephone appointments easier or more accessible for you to use?
      1. Do you get a set appointment time? If not, would you prefer this? Why?
3. Do you know if your GP practice offers videocall appointments? Is there a particular time or situation in which you would or wouldn’t use videocall appointments? If yes, why?
   1. Is there anything that could be done, or introduced, what would make videocall appointments easier or more accessible for you to use?
4. Other than those we have already discussed, are there any other positives/benefits or negatives/challenges, of using alternative platforms for appointments for you?

**Future use/training and support**

1. So thinking about how alternative platforms should be used going forward, in what situations do you think they are best used?
   1. How do you think alternative platforms could be delivered in the future to be more accessible and acceptable to more patients?
   2. Why do you think telephone/e-consults are increasing in use? Why do think that?
   3. Do you think patients should be given a choice about whether they get a call or a in-person appointment?
2. Do you think specific training is needed for GPs and nurses to deliver mental health appointments using these alternative platforms? If so, what do you think they need?
3. Finally, is there anything you want to talk about in relation to alternative platforms for appointments for anxiety and depression, or alternative platforms more generally?
