# Supplementary material for: Primary care practitioners’ and patients’ views on the benefits and challenges of remote consulting for anxiety and depression in UK general practice: a qualitative interview study
Source: BMJ Open. 2025 Jun 8;15(6):e093795. doi: 10.1136/bmjopen-2024-093795 (PMC12161304; doi:10.1136/bmjopen-2024-093795)
Supplement: Supplementary file 2 [file bmjopen-2024-093795.reviewer_comments.pdf]

PEER REVIEW HISTORY

BMJ Open publishes all reviews undertaken for accepted manuscripts. Reviewers are asked to complete a checklist review form and are provided with free text boxes to elaborate on their assessment. These free text comments are reproduced below.

ARTICLE DETAILS

Title (Provisional)

Primary care practitioners' and patients' views on the benefits and challenges of remote consulting for anxiety and depression in general practice: A qualitative interview study

Authors

Archer, Charlotte; Ting, Louise; Kessler, David; Wiles, Nicola; Turner, Katrina M

VERSION 1 - REVIEW

**Reviewer**	**1**
**Name**	**Stevenson, Fiona**
**Affiliation**	**UCL, Primary care and population sciences**
**Date**	**28-Oct-2024**
**COI**	**None**

This is a well written paper using interview data to explore the views of primary care practitioners and patients on the benefits and challenges of remote consulting for anxiety and depression in general practice. My comments are minor and aimed at encouraging further reflection to make the most of what appears to be a strong dataset.

There is limited data in the paper referring to e-consults, could the authors comment on this and reflect on the role of e-consults for common mental disorders?

Can the authors comment on the apparent contradiction that seem to be raised by this data of the importance of continuity of care for people experiencing mental distress, but at the same time concerns about responding rapidly in case patients self-harm, alongside the pressure on delivery of service, by GPs in particular, even when delivering remotely

In the discussion about training for consulting remotely for mental health consultations, how can this be distinguished from more general training for consulting remotely? Surely empathy is a skill required for more than just consulting about mental health concerns. Similarly, the discussion about whether patients are in a safe and appropriate space to be able to consult. The authors might find this reference useful in this regard Humphrey et al (2024) GP remote consultations with marginalised patients and the importance of place

during care: a qualitative study of the role of place in GP consultations DOI: https://doi.org/10.3399/BJGPO.2024.0050.

Overall, the authors present interesting data, however I think could have provided a higher level of interpretation reflecting across all the data they present to comment on the overall differences between the experiences of practitioners and patients in remote consultations for mental health concerns and in person consultations. The extract at the bottom of p11 indicates that the more transactional structure of remote consultations may limit disclosures, yet the summary in the discussion suggests remote consultations improve access to care. Can the authors unpick this in the discussion and reflect on the complexity of providing and accessing care particularly for mental health disorders.

**Reviewer**	**2**
**Name**	**Awan, Hassan**
**Affiliation**	**Keele University**
**Date**	**19-Mar-2025**
**COI**	**None**

Thank you for the opportunity to review this paper. This is an important topic considering anxiety and depression are significantly managed in primary care via remote consulting, without a research base exploring the impact of this. It is a well-conducted and well-written study and the findings are potentially practice-changing - as practicing GP I will consult differently after reading it.

Some minor suggestions are below

Abstract:

'Remote consultations may not be more time or cost-efficient for individuals with CMDs as risk is harder to assess.' This doesn’t follow from the findings as they seem to indicate an initial in-perspn appointment is important. Could the findings be made clearer to follow the conclusion?

There is a need to evaluate existing training to identify whether remote mental health care is included or should be incorporated in the future. What training is being referred to here?

Background:

Relevant literature included

Methodology

Thorough methodology described in details. Well done for having a PPI co-investigator!

The study included pharmacists and wellbeing coaches – they would potentially speak to people with CMDs from very different perspectives, for example risk assessment would be less of a priority. Have you looked at differences between practitioner perspectives?

Interviews took place only telephone or videocall – it would be helpful to have some reflexivity around how this may have influenced participants taking part. Would it bias towards those more comfortable with such methods?

Results

Figure 1 spelling mistake (paeient)

Text around quotes of not getting to the root of the problem could be expanded – what would the patients/GPs want to do in person that is different to get to the root of the problem?

Behavioural activation section feels under-developed – can this be expanded.

Discussion

Some important findings could be further mentioned in the discussion, such as prescribing more due to lower confidence managing risk.

Video consulting has not been mentioned much, appreciating because it is used little. Could the authors consider if it is an option that could be used more? Or not? And why.

To a clinical audience to make it more clinically relevant, could a top tips for remote consulting for CMDs be included?

**Reviewer**	**3**
**Name**	**Woods, Catherine**
**Affiliation**	**Oxford University, Primary Care**
**Date**	**21-Mar-2025**
**COI**	**None**

Overall, I thought your paper was clear and it adds an important contribution to the evidence base about the utility of remote consultations for people consulting in primary care with mental health problems. I agree that more work still needs to be done regarding evaluating remote modalities and training options for practitioners. Perhaps a next step of the research is also to diversify the patient population and clinician skill mix, as you largely recruited GPs and patients of a white ethnic origin. But for the purposes of this study, I think you met your objectives and it is an interesting read. Below are minor comments to consider when revising the manuscript:

First sentence of the manuscript (line 1) – could you include a better reference for this instead of the Murphy paper (which is about remote consulting)? And perhaps emphasise that these conditions are primarily managed in primary care because they suit/require continuity of care afforded from these services?

I think the background is overall well-referenced. You might want to highlight that your study is filling an evidence gap re evaluating the suitability of remote consultations for mental health problems specifically and in primary care – a lot of existing evidence is either pre-covid or across conditions and not MH. So this study is important.

P4 (line 56) – when you say you originally aimed to recruit GPs and nurses, it might be worth stating that you aimed to recruit (presumably) advanced nurse practitioners/prescribers, as they are dealing with MH problems more and more, but their role is not as well understood?

P5 (line 10) – why did you use postal consent forms rather than e-consent? This is more of a curiosity question, but also relevant considering the paper is about remote consulting (perhaps e-consent might be something to consider for a future study as more efficient?).

You might want to include a sentence about ethics approval in your methods section (even if just approval code from the relevant ethics committee).

Your data analysis section is easy to read and robust. Mention of 'deviant cases' is important but not always included, but it fits well here.

P6 – I would consider reporting the ethnicity of the practitioners too as it feels slightly odd to only report it for patients.

The findings are easy to read and well-justified with quotes.

P13 (lines 53-54) – your point "Patients and practitioners had little experience of receiving or providing care by videocall thus limiting our understanding of the benefits, challenges and facilitators of this mode of consultation" – I would include in brackets that (most) practices are not offering a high proportion of video-consultations, so it would be hard to get in-depth views on this anyway (so not as much of a limitation of your study).

VERSION 1 - AUTHOR RESPONSE

Reviewer: 1
Dr. Fiona Stevenson, UCL
Comments to the Author:
This is a well written paper using interview data to explore the views of primary care practitioners and patients on the benefits and challenges of remote consulting for anxiety and depression in general practice. My comments are minor and aimed at encouraging further reflection to make the most of what appears to be a strong dataset.

Thank you for your review of the manuscript and for your helpful comments.

1. There is limited data in the paper referring to e-consults, could the authors comment on this and reflect on the role of e-consults for common mental disorders?

Some of the themes also include data on e-consults, but these are more limited as practitioners and patients tended to talk about e-consults in a more generic way, rather than specific to mental health. We have now acknowledged and explained this early on in the findings (page 7, paragraph 1) by adding the following text:

Themes related to e-consults are those that are specific to CMDs, rather than generic for all health conditions.

2. Can the authors comment on the apparent contradiction that seem to be raised by this data of the importance of continuity of care for people experiencing mental distress, but at the same time concerns about responding rapidly in case patients self-harm, alongside the pressure on delivery of service, by GPs in particular, even when delivering remotely

There was a tension between continuity of care and a timely response, although this applies to all care, not just care delivered remotely. We have added a paragraph to the comparisons with existing literature section to discuss this:

There is also a contradiction in our data between the importance of continuity of care yet the need to be able to respond rapidly in situations where risk is high. This is challenging to resolve as patients' ability to see the same practitioner is likely dependent on service delivery pressures and the urgency of the problem, which is often closely linked to risk, further compounding this contradiction [24]…[page 13, paragraph 4]

3. In the discussion about training for consulting remotely for mental health consultations, how can this be distinguished from more general training for consulting remotely? Surely empathy is a skill required for more than just consulting about mental health concerns. Similarly, the discussion about whether patients are in a safe and appropriate space to be able to consult. The authors might find this reference useful in this regard Humphrey et al (2024) GP remote consultations with marginalised patients and the importance of place during care: a qualitative study of the role of place in GP consultations DOI: https://doi.org/10.3399/BJGPO.2024.0050.

We are aware of the focus on empathy within the 'Training in remote mental health consultations' theme of the results, and in the discussion, and we agree that empathy is a key skill for all types of consultations. Interviewees in our study emphasised the importance of conveying empathy in order to encourage disclosure and assess risk or to ask the right questions to assess whether the patient is at risk, despite not being able to see them. When practitioners did not convey empathy, patients suggested they may withhold information, leading to under recognised risk of self-harm/suicidality. It is the relationship between the demonstration of empathy and subsequent disclosure/risk

assessment that is crucial in a mental health consultation. We have added the following text in the relevant theme (Training in remote mental health consultations) to make this clearer (new text underlined):

Similarly, patients also talked about the need for practitioner training in conveying empathy over the telephone. They viewed empathy as a key facilitator in encouraging disclosure around CMDs, leading to a more accurate risk assessment, as well as the need for practitioners to develop active listening skills, so they did not interrupt the patient but listened carefully and reflected back to them. [page 11, paragraph 4]

We also agree that the need for a safe space is not specific mental health conditions and can be applied more broadly. However, we have included it in this paper as it was pertinent to our interviewees and feels particularly relevant to mental health (whilst also being relevant for general health). Thank you for sharing the Humphrey paper, we have added this to the discussion section:

In our study, in-person consultations at the practice were considered key in providing a safe and private space to discuss concerns. This is consistent with other research exploring the role of place in GP consultations [27]. Not all patients are able to access appropriate private spaces for remote consults and this can negatively impact on their experiences of care, thus creating inequities. It was noted that such spaces are particularly important for those discussing sexual or mental health problems [27]. [page 14, paragraph 2]

4. Overall, the authors present interesting data, however I think could have provided a higher level of interpretation reflecting across all the data they present to comment on the overall differences between the experiences of practitioners and patients in remote consultations for mental health concerns and in person consultations. The extract at the bottom of p11 indicates that the more transactional structure of remote consultations may limit disclosures, yet the summary in the discussion suggests remote consultations improve access to care. Can the authors unpick this in the discussion and reflect on the complexity of providing and accessing care particularly for mental health disorders.

We have added a paragraph into the discussion to add some further commentary around this area:

Many of our interviewees said that remote modes increased access to care, and this supports findings by NHS England [5]. Unfortunately, increased provision of care by practitioners does not always equate to increased engagement by those accessing care, with some patients sharing their experience of limiting how much information they disclosed over the telephone, potentially due to the transactional nature and not wanting to take up practitioner time. A recent briefing highlights it is important to consider access to healthcare as more than just the provision of appointments, it is also

about broader factors, such health literacy, barriers to reach services, and how people identify themselves as candidates for medical attention [23]. [page 13, paragraph 4]

Reviewer: 2
Dr. Hassan Awan, Keele University
Comments to the Author:
Thank you for the opportunity to review this paper. This is an important topic considering anxiety and depression are significantly managed in primary care via remote consulting, without a research base exploring the impact of this. It is a well-conducted and well-written study and the findings are potentially practice-changing - as practicing GP I will consult differently after reading it. Some minor suggestions are below

Thank you for your time taken to review this paper, and for your helpful comments.

5. Abstract: 'Remote consultations may not be more time or cost-efficient for individuals with CMDs as risk is harder to assess.' This doesn't follow from the findings as they seem to indicate an initial in-person appointment is important. Could the findings be made clearer to follow the conclusion?

We have added the following text to the findings to make the link to our conclusion clearer (new text underlined):

Challenges included practitioners finding it hard to assess risk which lengthened consultation duration or led to further contact, increasing practice workload, and… [page 2, paragraph 4]

6. There is a need to evaluate existing training to identify whether remote mental health care is included or should be incorporated in the future. What training is being referred to here?

There is self-directed e-training offered by the NHS on remote consultations (https://www.e-lfh.org.uk/programmes/remote-consultations/) and by RCGP on Telephone consultation and triage skills (https://www.rcgp.org.uk/learning-resources/courses-and-events/consulting-skills), which practitioners are able to complete. We have made this clearer in the abstract:

There is a need to evaluate existing training on delivering remote consultations to identify whether remote mental health care is included or should be incorporated in the future. [page 2, paragraph 5]

7. Background: Relevant literature included
Methodology: Thorough methodology described in details. Well done for having a PPI

co-investigator! The study included pharmacists and wellbeing coaches – they would potentially speak to people with CMDs from very different perspectives, for example risk assessment would be less of a priority. Have you looked at differences between practitioner perspectives?

Yes we did consider each practitioners role during thematic analysis. The main difference was related to risk around prescribing, where AHPs were less confident in prescribing psychotropics if they had not seen the patient in person [see findings>loss of information over the telephone]. There were also some differences relating to the theme of behavioural activation, and we have expanded on this in that section (new text underlined):

Some practitioners suggested that for patients who were anxious to leave home, attending in-person could be part of encouraging the patient to take control of their mental health and set achievable goals. This point was primarily made by the AHPs interviewed, and by those who had patients with a chronic CMD with whom they had developed a good doctor-patient relationship with during long-term remote care. [page 12, paragraph 4]

8. Interviews took place only telephone or videocall – it would be helpful to have some reflexivity around how this may have influenced participants taking part. Would it bias towards those more comfortable with such methods?

It is possible that we interviewed those who were more comfortable with remote modes, although we did capture a range of views in the dataset. We have acknowledged this potential limitation in the discussion (new text underlined):

Likewise, patients and practitioners were self-selecting in terms of being individuals who responded to the invitation, which stated the research was focused on remote methods (telephone, video, e-consult) for consulting in general practice and that the interviews were being held by telephone or videocall. It is possible that those interviewed were individuals who had strong preferences for or against remote consultations and were comfortable being interviewed remotely. [page 13, paragraph 1]

Results

9. Figure 1 spelling mistake (patient)

Thank you - this has been corrected.

10. Text around quotes of not getting to the root of the problem could be expanded – what would the patients/GPs want to do in person that is different to get to the root of the problem?

We have expanded this section to include practitioner/patient suggestions on this:

There was a sense that telephone calls were less comprehensive without the visual cues, so skilled questioning to elicit information on aetiology and severity of the CMD, alongside conveying understanding and reassurance were pertinent when using this mode. [page 9, paragraph 11]

11. Behavioural activation section feels under-developed – can this be expanded.

We have expanded on the theme of behavioural activation (new text underlined):

Some practitioners suggested that for patients who were anxious to leave home, attending in-person could be part of encouraging the patient to take control of their mental health and set achievable goals. <u>This point was primarily made by the AHPs interviewed, and by those who had patients with a chronic CMD with whom they had developed a good doctor-patient relationship with during long-term remote care.</u> [page 12, paragraph 4]

Discussion

12. Some important findings could be further mentioned in the discussion, such as prescribing more due to lower confidence managing risk.

We have added the following to the discussion summary and comparison with existing literature:

… and some are more likely to prescribe medication if they were not confident in their ability to assess patient risk when consulting remotely. [page 12, paragraph 7]

…Some practitioners in our study suggested they might be more likely to prescribe over the telephone if they were concerned about risk. This is not in line with analyses conducted prior to the pandemic using secondary care electronic health records, which did not find an increase in psychiatric prescribing [26]. However, there is some evidence that remote consultations have higher doses of anxiolytics [27] and increased antibiotic prescribing rates [28], suggesting mode may be a factor in prescribing decisions. [page 14, paragraph 6]

13. Video consulting has not been mentioned much, appreciating because it is used little. Could the authors consider if it is an option that could be used more? Or not? And why.

Interviewees in our study did not have a preference for videocall due to the potential technical problems and digital literacy needed by patients to be able to use them effectively. Despite none of the patients we interviewed having experience of using

videocalls for mental health, many were indifferent to the option, suggesting they might as well just telephone, or go into the practice if the GP wanted to 'see' them. None of the interviewee reasons given for or against videocalls were specific to mental health, so we have not focused on these in the paper. We also note that a paper by Greenhalgh et al (*2022- Why do GPs rarely do video consultations? qualitative study in UK general practice. BJGP*) covers this topic comprensively. We have expanded on this area in our limitations section (new text underlined):

Patients and practitioners had little experience of receiving or providing care by videocall thus limiting our understanding of the benefits, challenges and facilitators of this mode of consultation. Future studies could seek to interview primary care patients who have used videocalls for CMDs. That said, most practices do not offer video consultations frequently, so it would be challenging to seek in-depth views on this mode [16]. [page 13, paragraph 1]

14. To a clinical audience to make it more clinically relevant, could a top tips for remote consulting for CMDs be included?

Thank you for this thoughtful suggestion. Due to the limitations of the study and the focus mainly on telephone consultations, we do not think it appropriate to provide a top tip list for remote consultations for CMDs. However, within the conclusion section, we have included the following text which indicates what practitioners (and practice managers) might want to consider when conducting remote consultations with individuals with a CMD:

It is important to offer in-person appointments, especially when patients initially seek help for anxiety or depression, and are unknown to the practitioner. Ideally practices should provide a narrower window for a telephone call…it is important for practitioners to convey empathy, ask questions that encourage disclosure, and demonstrate active listening. [page 14, paragraph 4]

Reviewer: 3
Dr. Catherine Woods, Oxford University
Comments to the Author:
Overall, I thought your paper was clear and it adds an important contribution to the evidence base about the utility of remote consultations for people consulting in primary care with mental health problems. I agree that more work still needs to be done regarding evaluating remote modalities and training options for practitioners. Perhaps a next step of the research is also to diversify the patient population and clinician skill

mix, as you largely recruited GPs and patients of a white ethnic origin. But for the purposes of this study, I think you met your objectives and it is an interesting read.

Thank you for your time taken to review our manuscript, and for your helpful comments.

Below are minor comments to consider when revising the manuscript:

15. First sentence of the manuscript (line 1) – could you include a better reference for this instead of the Murphy paper (which is about remote consulting)? And perhaps emphasise that these conditions are primarily managed in primary care because they suit/require continuity of care afforded from these services?

The first sentence now reads as (new text underlined, and reference changed):

Anxiety and depression are managed in primary care with GP practices best placed to offer the continuity of care that these conditions often require [1 - Kroenke et al, 2007)]. [page 3, paragraph 1]

16. I think the background is overall well-referenced. You might want to highlight that your study is filling an evidence gap re evaluating the suitability of remote consultations for mental health problems specifically and in primary care – a lot of existing evidence is either pre-covid or across conditions and not MH. So this study is important.

We have added the following sentence to the background section to make this evidence gap clear:

Much of the existing evidence on remote consulting was either conducted prior to the pandemic or is focused across all conditions and not specific to mental health. [page 3, paragraph 4]

17. P4 (line 56) – when you say you originally aimed to recruit GPs and nurses, it might be worth stating that you aimed to recruit (presumably) advanced nurse practitioners/prescribers, as they are dealing with MH problems more and more, but their role is not as well understood?

We have expanded this section as per you suggestion (new text underlined):

Initially the research team aimed to recruit GPs and practice nurses, as they both have a role in supporting primary care patients with mental health. [page 4, paragraph 1]

18. P5 (line 10) – why did you use postal consent forms rather than e-consent? This is more of a curiosity question, but also relevant considering the paper is about remote consulting (perhaps e-consent might be something to consider for a future study as more efficient?).

They were postal invitations with response forms rather than consent forms. Consent was collected verbally prior to the interview. We used postal invitations as most of the practices we purposefully sampled were still corresponding with their patients by post, rather than by email. However, in future studies we will consider offering practices the option of inviting their patients by email (in addition to post), in order to maximise potential participation and efficiency.

19. You might want to include a sentence about ethics approval in your methods section (even if just approval code from the relevant ethics committee).

We have added the following text at the start of the methods section:

Ethical approval was provided by South West - Frenchay Research Ethics Committee (REC reference: 22/SW/0136). [page 3, paragraph 5]

20. Your data analysis section is easy to read and robust. Mention of 'deviant cases' is important but not always included, but it fits well here. P6 – I would consider reporting the ethnicity of the practitioners too as it feels slightly odd to only report it for patients.

We have added practitioner ethnicity information to Table 2.

21. The findings are easy to read and well-justified with quotes. P13 (lines 53-54) – your point "Patients and practitioners had little experience of receiving or providing care by videocall thus limiting our understanding of the benefits, challenges and facilitators of this mode of consultation" – I would include in brackets that (most) practices are not offering a high proportion of video-consultations, so it would be hard to get in-depth views on this anyway (so not as much of a limitation of your study).

Thank you for this suggestion, we have added the below sentence:

That said, most practices – do not offer video consultations frequently, so it would be challenging to seek in-depth views on this mode [16]. [page 13, paragraph 1]

VERSION 2 - REVIEW

**Reviewer**	**1**
**Name**	**Stevenson, Fiona**
**Affiliation**	**UCL, Primary care and population sciences**
**Date**	**07-May-2025**
**COI**	

Thank you to the authors for addressing the comments so comprehensively - I have no further comments

**Reviewer**	**2**
**Name**	**Awan, Hassan**
**Affiliation**	**Keele University**
**Date**	**01-May-2025**
**COI**	

All reviewer comments have been adequately responded to thank you, no further comments

**Reviewer**	**3**
**Name**	**Woods, Catherine**
**Affiliation**	**Oxford University, Primary Care**
**Date**	**30-Apr-2025**
**COI**	

Well done on a speedy revision and attending to all reviewer comments. I think the paper is an important contribution and I will be citing it in the future.
